# Five new species of dictyostelid social amoebae (Amoebozoa) from Thailand

**DOI:** 10.1186/s12862-018-1328-5

**Published:** 2018-12-22

**Authors:** Eduardo Vadell, James C. Cavender, John C. Landolt, Allison L. Perrigo, Pu Liu, Steven L. Stephenson

**Affiliations:** 1 Escuela de Bioquimica, Departamento de Biologia, J. F. Kennedy University, Buenos Aires, Argentina; 2 Departmental of Environmental and Plant Biology, Ohio University, Athens, OH 45701 USA; 3 Department of Biology, Shepherd University, Shepherdstown, West Virginia 25443 USA; 4 Gothenburg Global Biodiversity Centre, Gothenburg, Sweden; 5 Department of Biological and Environmental Sciences, University of Gothenburg, Gothenburg, Sweden; 6 Engineering Research Center of Chinese Ministry of Education for Edible and Medicinal Fungi, Jilin Agricultural University, Changchun, 130118 China; 7 Department of Biological Sciences, University of Arkansas, Fayetteville, AR 72701 USA

**Keywords:** Biodiversity, Cellular slime molds, Dictyostelids, Forest soils, Southeast Asia, Amoebozoa, Protist taxonomy, *Cavenderia*

## Abstract

**Background:**

Dictyostelid cellular slime molds (dictyostelids) are common inhabitants of the soil and leaf litter layer of fields and forests, along with animal dung, where they feed mostly on bacteria. However, reports on the species diversity of dictyostelids in South Asia, particularly Thailand, are limited. The research reported in this paper was carried out to increase our knowledge of the species diversity of this group of organisms in northern Thailand.

**Results:**

Forty soil samples were collected at four localities in northern Thailand to assess the species richness of dictyostelids. These samples yielded five dictyostelid isolates that were not morphologically consistent with any described species. Based on molecular signatures, all five of these isolates were assigned to the family Cavenderiaceae, genus *Cavenderia.* All five share a number of morphological similarities with other known species from this family. The new taxa differ from previously described species primarily in the size and complexity of their fruiting bodies (sorocarps). This paper describes these new species (*Cavenderia aureostabilis*, *C*. *bhumiboliana*, *C*. *protodigitata*, *C*. *pseudoaureostipes*, and *C*. *subdiscoidea*) based on a combination of morphological characteristics and their phylogenetic positions.

**Conclusions:**

At least 15 taxa of dictyostelids were obtained from the four localities in northern Thailand, which indicates the high level of species diversity in this region. Five species were found to be new to science. These belong to the family Cavenderiaceae, genus *Cavenderia*, and were described based on both morphology and phylogeny.

## Background

Dictyostelid cellular slime molds (dictyostelids) have been reported from numerous localities throughout the world, but there are relatively few records from Southeast Asia. Furthermore, their taxonomy is problematic and poorly understood, and key higher-level taxa have only recently been delimited based on molecular signatures [1]. This major step revised the taxonomy of the dictyostelids, which was originally based on a three-genus system based which used fruiting body formation and branching patterns of the multicellular stage in the life cycle to delimit genera [2]. Hagiwara’s taxonomy [3] introduced other morphological features such as spore shape, size and granulation pattern, aggregation pattern, size and growth habit of sorocarps, and the shape of the sorophore tip and base. This greatly improved species level identifications and descriptions.

Dictyostelids are ubiquitous and most frequently found in the uppermost layers of soils [4]. However, despite their ubiquity, the amoebae are not readily visible in soil samples, and DNA techniques have not focused on methods that will recover representatives of this taxon. To recover dictyostelids, soils must be cultured under laboratory conditions to confirm their presence. The procedure used, referred to as the “Cavender Method” [5] is relatively easy to carry out and has produced significant results over a period of more than 50 years. Moreover, it is possible to obtain both quantitative and qualitative data for the clones of fruiting bodies which appear in these primary isolation cultures. Unidentified fruiting bodies are transferred to two-membered culture with *Escherichia coli* and further study of morphological features eventually leads to a species-level diagnosis. Although global knowledge of dictyostelid diversity is still relatively limited, we do know a great deal about their global distribution [6]. Progress has been made so that the number of species described has more than doubled since the printing of Raper’s monograph [2] and has been sufficient for phylogenetic studies to form a new taxonomy based on molecular as well as morphological techniques. An accurate knowledge of dictyostelid diversity is a key to any subsequent work on the group, including ecological and functional studies. As such, this paper provides an example of the kind of information that can be gathered from a typical distributional study using both morphological and molecular techniques.

In the 1970s, Cavender [7, 8] carried out a survey for dictyostelids in the forests of Southeast Asia, including sites in Thailand and mainland Malaysia, as well as in the Philippines and the Indonesian island of Java. Sixteen different species were recovered, including four new species: *Dictyostelium intermedium* Cavender, *D*. *multistipes* Cavender [now *Cavenderia multistipes* (Cavender) S. Baldauf, S. Sheikh & Thulin)], *D*. *bifurcatum* Cavender [now *C. bifurcata* (Cavender) S. Baldauf, S. Sheikh & Thulin] and *Actyostelium subglobosum* Cavender. In that study, Thailand was represented by three collecting sites, and these yielded a total of nine different species.

This early work demonstrated that species diversity of the dictyostelids is high in Southeast Asia and that many species remain to be described. Herein, initial results from a follow-up survey of the dictyostelids of Thailand are reported. Five species from four new sampling sites in Thailand are described herein. These are *Cavenderia aureostabilis*, *C*. *bhumiboliana*, *C*. *protodigitata*, *C*. *pseudoaureostipes*, and *C*. *subdiscoidea.*

## Results

**Fig. 1:**
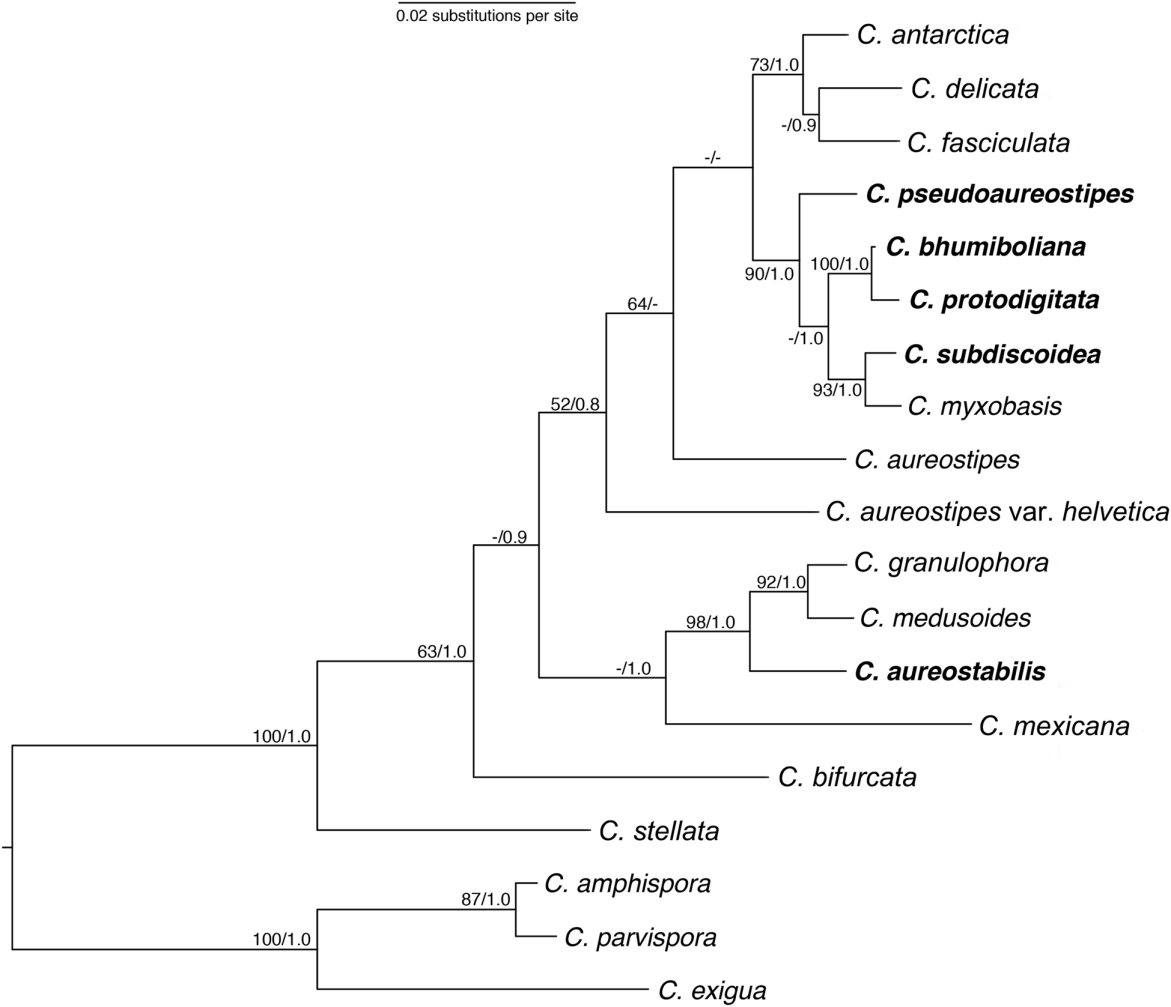
Phylogeny of a subset of closely related species in the genus *Cavenderia* (Dictyostelia) indicating the phylogenetic position of the five newly described species: *Cavenderia pseudoaureostipes, C. bhumiboliana, C. protodigitata, C. subdiscoides*, and *C. aureostabilis*. The tree was derived by Bayesian analysis of the ribosomal small subunit (SSU). Names in bold are the new species described herein. Maximum likelihood bootstrap support (BS) over 50% and Bayesian inference posterior probabilities (PP) over 0.70 are indicated on the branches, to the left and right of the slash, respectively. The phylogeny is rooted according to Schaap et al. (2006)

The samples collected in northern Thailand yielded a total of at least 15 taxa. These included *Acytostelium* sp., *Coremiostelium polycephalum* (Raper) S. Baldauf, S. Sheikh, *Dictyostelium giganteum* B.N. Singh, *D*. *mucoroides* Bref., *D. purpureum* Olive, *D*. *sphaerocephalum* (Oudem.) Sacc. & Marchal, *Hagiwaraea lavandula* (Raper & Fennell) S. Baldauf, S. Sheikh & Thulin, *Heterostelium candidum* (H. Hagiw.) S. Baldauf, S. Sheikh & Thulin, *H. pallidum* (Olive) S. Baldauf, S. Sheikh & Thulin, *Polysphondylium violaceum* Bref. and *Tieghemostelium lacteum* (Tiegh.) S. Baldauf, S. Sheikh & Thulin. In addition, five new species were identified among the isolates recovered and are described herein. A summary comparison of the morphological features of the five isolates is provided in Table 1, and the phylogenetic relationships among the new taxa and closely related species are presented in Fig. 1.

**Table 1:** Summary data on the new species of *Cavenderia* described herein. *C*. *aureostipes* is included for comparison (**in bold**)

Species	Spores	Sori	Yellow Pigmentation	Size	Branches	Agg	Clusters	Slime	Base	Migr	Elevation (m)
*Cavenderia aureostabilis*	M-L	M	Intense	L	0	Radial	None	++	Disk	++++	1000
*C*. *bhumiboliana*	L	S	Fades	S	0–2	Mounds	Few	+++	Clavate-digitate	+	2500
*C*. *protodigitata*	S-M	S	Fades	S	0–2	Mounds	Few	+++	Clavate-digitate	+	2500
*C*. *pseudoaureostipes*	M	L	Intense	M-L	> 20	PV	Few	+	Digitate-round	+++	800
*C*. *subdiscoidea*	M	S-L	Intense	L	4–20	PV	Few	+	Disk-round	+++	800
***C*** **.** ***aureostipes***	**M**	**M**	**Strong**	**M**	> 20	**PV**	**Few**	**+**	**Round-irregular**	**+**	**300**

Spores: *M* median (most common range: 5.5–7.5 × 2.5–3.5 μm), *L* large (most common range: 6.5–9 × 3–5 μm), *S* small (most common range: 4.5–5.5. × 2–3 μm). Sori: *S* small (most common range: 20–100 μm), *M* median (commonest range: 80–150 μm), *L* large (commonest range: 150–250 μm). Sorocarp Size: *S* small (commonest range: 0.2–3 mm), *M* median (commonest range: 3–5 mm), *L* large (most common range: 5–8 mm), *Agg* aggregation, *PV Polysphondylium violaceum* type. Slime: + = present, ++ = abundant, +++ = very abundant. Clusters: None = 0-to rarely 2–4 sorognens per mound, Few = frequently 2–5 sorogens per mound/pseudoplasmodium, Some = frequently 4–8 sorogens, Migr = pseudoplamodia migration: + = microns to few millimeters (mm) (0.2 to 2 (− 5) mm), +++ = 5–10 mm, ++++ = more than 10 mm

### Phylogeny

Molecular data from the ribosomal small subunit (SSU) further supports the placement of all five new taxa in the genus *Cavenderia* (Fig. 1). All five taxa have both of the molecular signatures characteristic of the genus in SSU Regions I and K [1]. *Cavenderia aureostabilis* is most closely related to *C. granulophora* (Vadell et al.) S.Baldauf, S.Sheikh & Thulin and *C. medusoides* Vadell, M.T. Holmes & Cavender*,* based on the SSU data, and together with these two species form a sister clade to *C. mexicana* (Cavender et al.) S.Baldauf, S.Sheikh & Thulin. The four other new species form a well-supported clade (90BS/1.0PP) together with *C. myxobasis* (Cavender, Vadell, J.C. Landolt & S.L. Stephenson) S. Baldauf, S. Sheikh & Thulin. All these species are very similar molecularly and spatially co-occur in nature. The SSU is highly conserved in many dictyostelid taxa, and in many cases cannot be relied on for species-level differentiation [9–11], for this reason, their morphological characteristics represent the primary justification for differentiation.

### Taxonomy

**Fig. 2:**
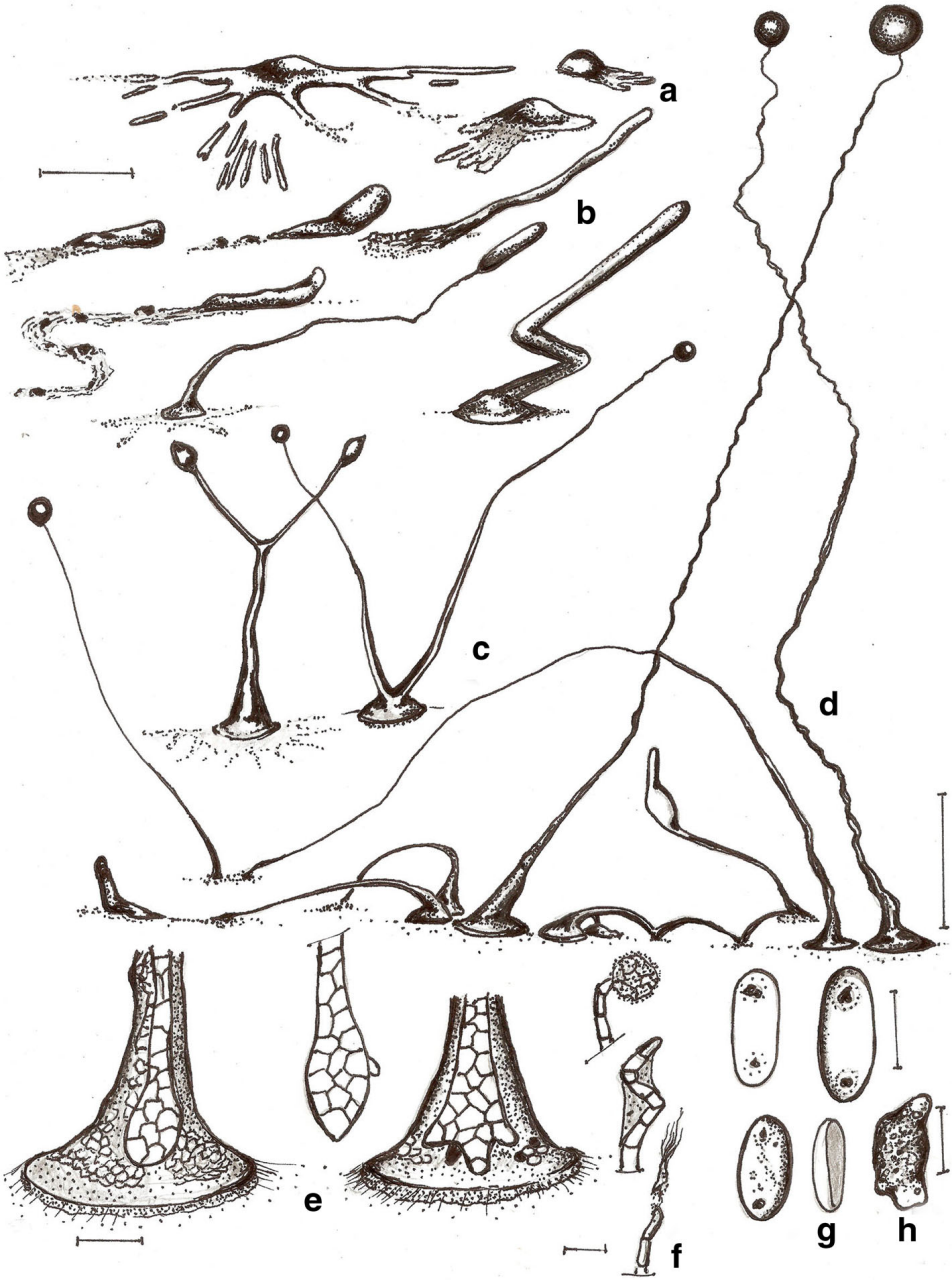
*Cavenderia aureostabilis* (TH10B). **a** Ample large streamed radiate aggregation with thin streams, separately, some brushy streams (left), smaller aggregations with shorter sided lobed streams (right). **b** Early highly migrating sorogens that leave traces of cells and slime, the tallest with stalk formation at its end (above, left to right), solitary late sorogen with a disk base and traces of early streams (non-migratory, below, left), early late sorogen that changes direction while migrating, becomes very elongated (below, right). **c** Late bifurcate sorogen with its ample cone to bell-shaped disk base (left), two young small sorocarps sharing a disk base (right). **d** Two clustered collapsed sorocarps, one continues a stoloniferous migration and the other not (left), firstly slender to weaving solitary unbranched sorocarp, well directed (center, left), a common stoloniferous habit of a late sorogen (center), two solitary unbranched mature sorocarps, one collapses and refruits, the other curved and weaving when aged (right). **e** Round base within a dense cone to bell-shaped regular matrix of slime that ends as an expanded prominent and regular disk, a cushion of differential slime below the base is attached to the substrate by thin inconspicuous fibers of sheath, the disk progressively filled in with small polygonal cells that covers all the body mass (left), clavate base with a protruding cell, the disk is omitted (center), shortly digitate base within the bell- to disk-shaped slime structure (right). **f** Simple one-celled tip holding a mass of rather undifferentiated cells and slime (above), curved simple tip with abundant dense slime (center), flexuous multi-piliform tip, all terminal cells are small. **g** Large narrow elliptical spores with irregular large consolidated PG, halos present, with vacuoles and small granules dispersed, a spore-cage (below, right). **h** Myxamoeba with multiple small-medium vacuoles. Scale bars: **a**, **b**: 300 μm; **c**, **d**: 0.5 mm; **e**: 25 μm; **f**: 10 μm; **g**: 6 μm; **h**: 10 μm

**Fig. 3:**
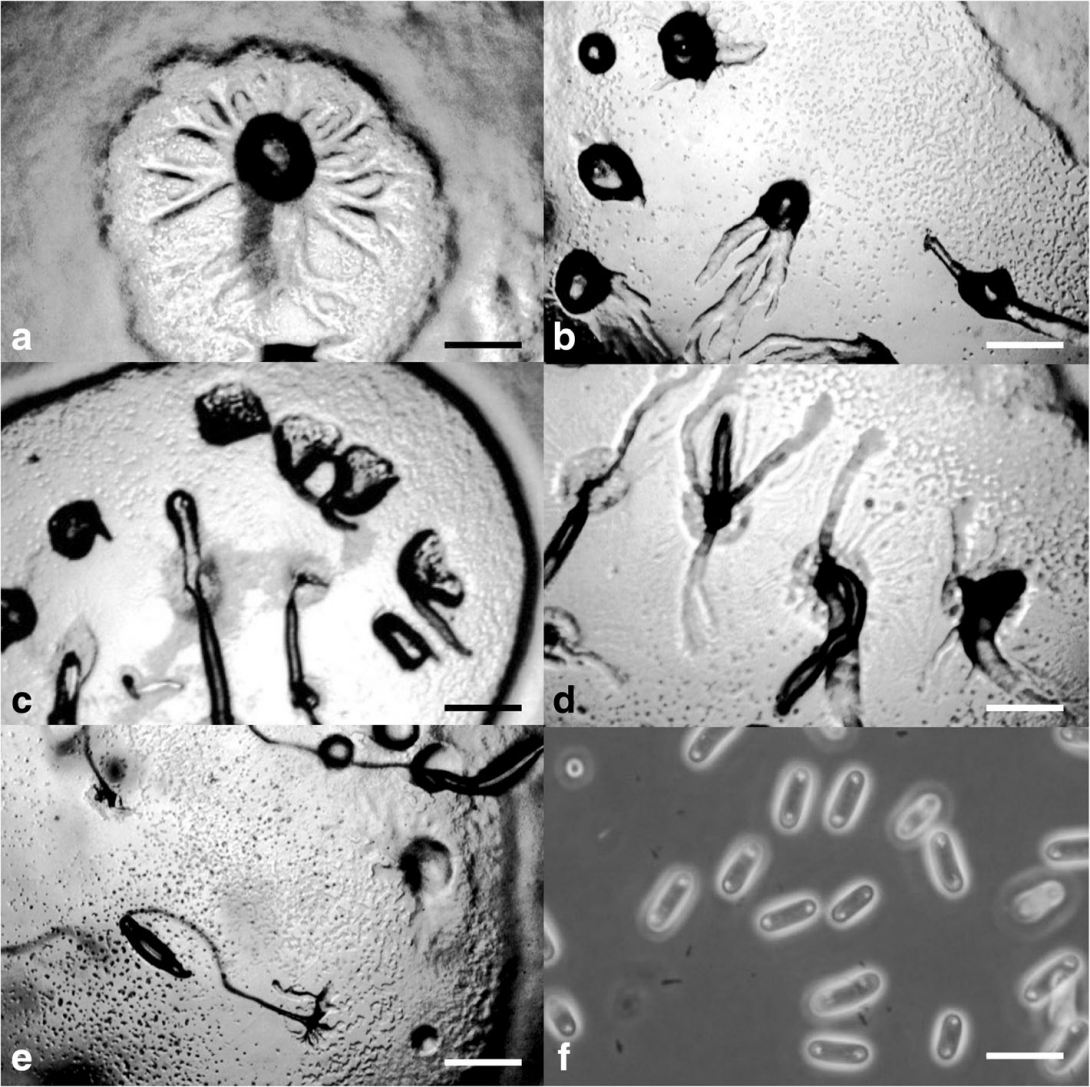
*Cavenderia aureostabilis* (TH10B). **a** Aggregations with radial streams. **b** Streaming aggregations. **c** Older aggregations with an unusual crinkled appearance and developing sorogens. **d** Aggregations producing single pseudoplasmodial sorogens. **e** Developing sorocarp. **f** Somewhat elongated, elliptical spores with consolidated polar granules. Scale bars: **a**–**e**: 225 μm; **f**: 10 μm

***Cavenderia aureostabilis*** Vadell, Cavender, J.C. Landolt, A.L. Perrigo, P. Liu & S.L. Stephenson, *sp. nov.* — MycoBank: MB827592; Figs. 2 and 3.

**Etymology***.* The name refers to the prominent yellow pigmentation.

**GenBank Accession Number**. MH745571.

**Culture examined.** Thailand, Mushroom Research Center, montane tropical forest, 128 Moo3, Bahn Pa Dheng, T. Pa Pae, A. Mae Taeng, Chiang Mai 50,150, 19°07.200’ N, 98°44.044′ E, isolated from a sample collected by Stephenson in January 2010, Landolt TH10B (Holotype), Ex–Landolt TH10B deposited in the Dicty Stock Center at Northwestern University (No. DBS0350787).

**Diagnosis. Sorocarps** solitary, mostly prone to decumbent, sigmoid to tortuous, stoloniferous 1–4 times and highly migratory, 2.0–5.5 mm long. **Sorophore** slender to curved, lower section irregular, granulated, with abundant supportive cells alongside, becoming irregularly tapered from base to tip, intensely yellow at first but the color soon fades to a stable and persistent lighter shade of yellow. Bases round to clavate, 1–8 celled (10–55 μm), with one or more enlarged protruding cells, these sometimes form crampons (Fig. 2e). Base resting on large discoid cushion (Fig. 2b, c) of dense granular slime that is gel-like, yellowish (80–120 μm diam.). Tips unfinished capitate (15–30 μm), may also end as a curved hook-shaped line of small irregular cells and/or be flexuous multi-piliform or attached to a sphere of rather undifferentiated small cells and slime (20–30 μm) (Fig. 2f). Subterminal segment consisting of one tier of cells and regular in structure. Sometimes the upper and lower sorocarp are bifid (base and terminus) (Fig. 2c). **Sori** globose, slightly yellow to hyaline, sticky, (60–)90–150(− 180) μm diam. **Spores** elliptical-oblong, large, regular, (6–)7–8.5(− 10) × 3–4(− 5) μm, with consolidated irregular polar granules (PG), not always round and many times surrounded by a halo, with a heterogeneous content (Fig. 2g). Spores do not germinate immediately. **Aggregations** large, radiate, streams (Fig. 3a) adopt brush shapes, becoming discontinuous. Streams also persist until the late sorogen stage. Small early sorogens (100–300 μm) emerge from the center of a pseudoplasmodium and may migrate freely. Late sorogens very elongated, tube-like (Figs. 2b and 3d), these collapse easily and continue migrating. Small blocky pseudoplasmodia may merge and rise up together. **Myxamoebae** large, yellow, slow, 8–15 × 10–18 μm, with many small to medium vacuoles and dark granules (Fig. 2h).

**Notes.** Late, long sorogens change directions, then curve, collapse and rise up again in a typical slow movement until settled. Individual myxamoebae continually creep up immature to mature sorocarps and remain as stalk supporters, then accumulate at a bend in the stalk or form a continuous mass of companion cells all along the stalk to the terminus. Some distant myxamoebae adopt a slug shape and merge as separate streams far away from the main aggregation. The lower sorophore with a cushion of dense slime has the appearance of a conical bell on more or less defined disk. The cultures do not endure long after cultivation and soon collapse. Most spores from collapsed sori germinate after a variable period of dormancy, the length depending upon the culture conditions. The slime matrix at the tip of the sorophore is hyaline with dark granules and differs in density and granular content from the base. The matrix and the sheath are very strong. The spore capsule splits longitudinally. Some myxamoebae round up close to the base. This species resembles *C. medusoides* but differs primarily in the larger size of the spores, the more distinct larger and irregular PG, a more constant median height and unbranched habit, more variable tips which are less defined as cellular capitate, smaller aggregations (600–1500 μm vs. 100–300 μm), a primary early to late sorogen that changes direction drastically and then, afterwards, begins migrating for several days, an expanded more conical basal sorophore terminus (bell-shaped) that rests on a disk-like structure.

**Fig. 4:**
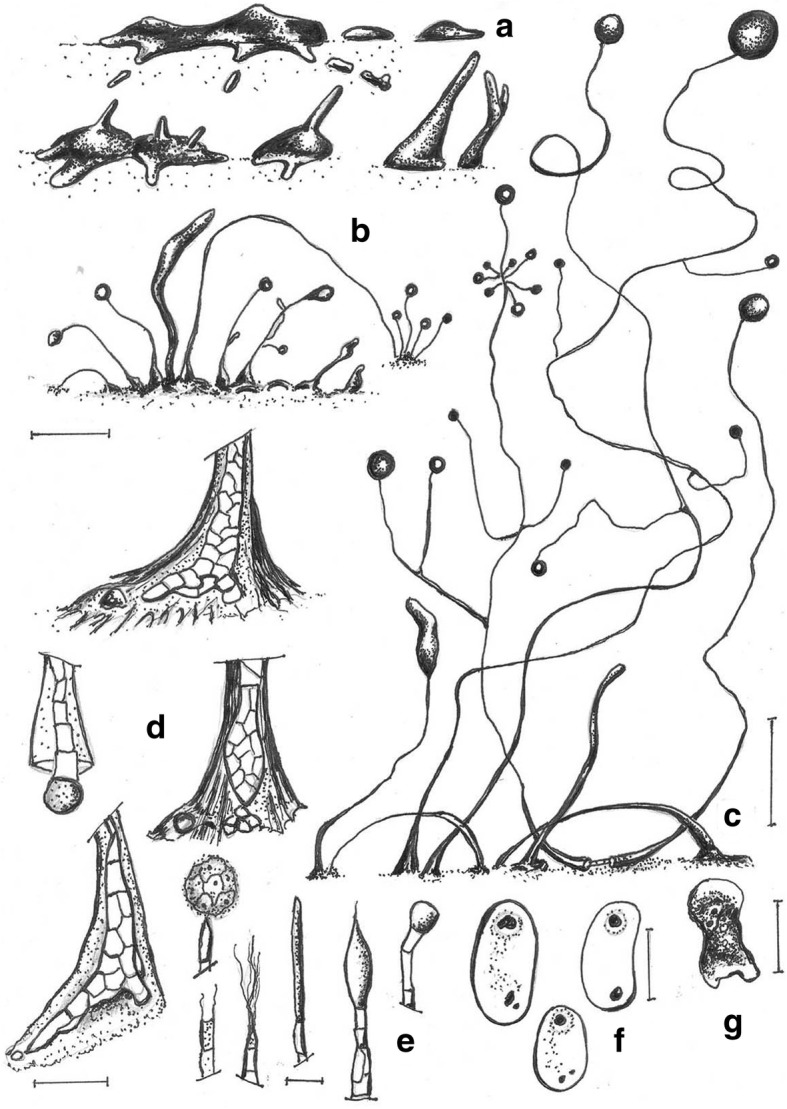
*Cavenderia bhumiboliana* (TH11CX). **a** Late *violaceum*-type aggregations, with irregular mounds or short thick streams. **b** Clustered early sorogens (above, left), solitary early sorogens (above, right), a crowded cluster of early and late sorogens, the mature central sorocarp has collapsed and its sorus has refruited (below). **c** Group of very curved sorocarps, two solitary but proximal fruiting bodies with few or no branches (left), two base-joined sorocarps in a cluster and a proximal one with stoloniferous habit (right). **d** Bases round to plane with sharp digitations (above), a single celled base and a clavate base with cells below the base (center), a shortly digitate base with sharp terminal cell (below). **e** Six variable tips, flexuous with piliform ends, one celled holding a mass of mucilage and rather undifferentiated cells, a thin elongated flexuous cell, an ampulla piliform cell and a short capitate single cell. **f** Broad elliptical regular spores with irregular consolidated PG. **g** Myxamoebae. Scale bars: **a,b**: 300 μm; **c**: 0.5 mm; **d**: 20 μm; **e**: 10 μm; **f**: 6 μm; **g**: 10 μm

**Fig. 5:**
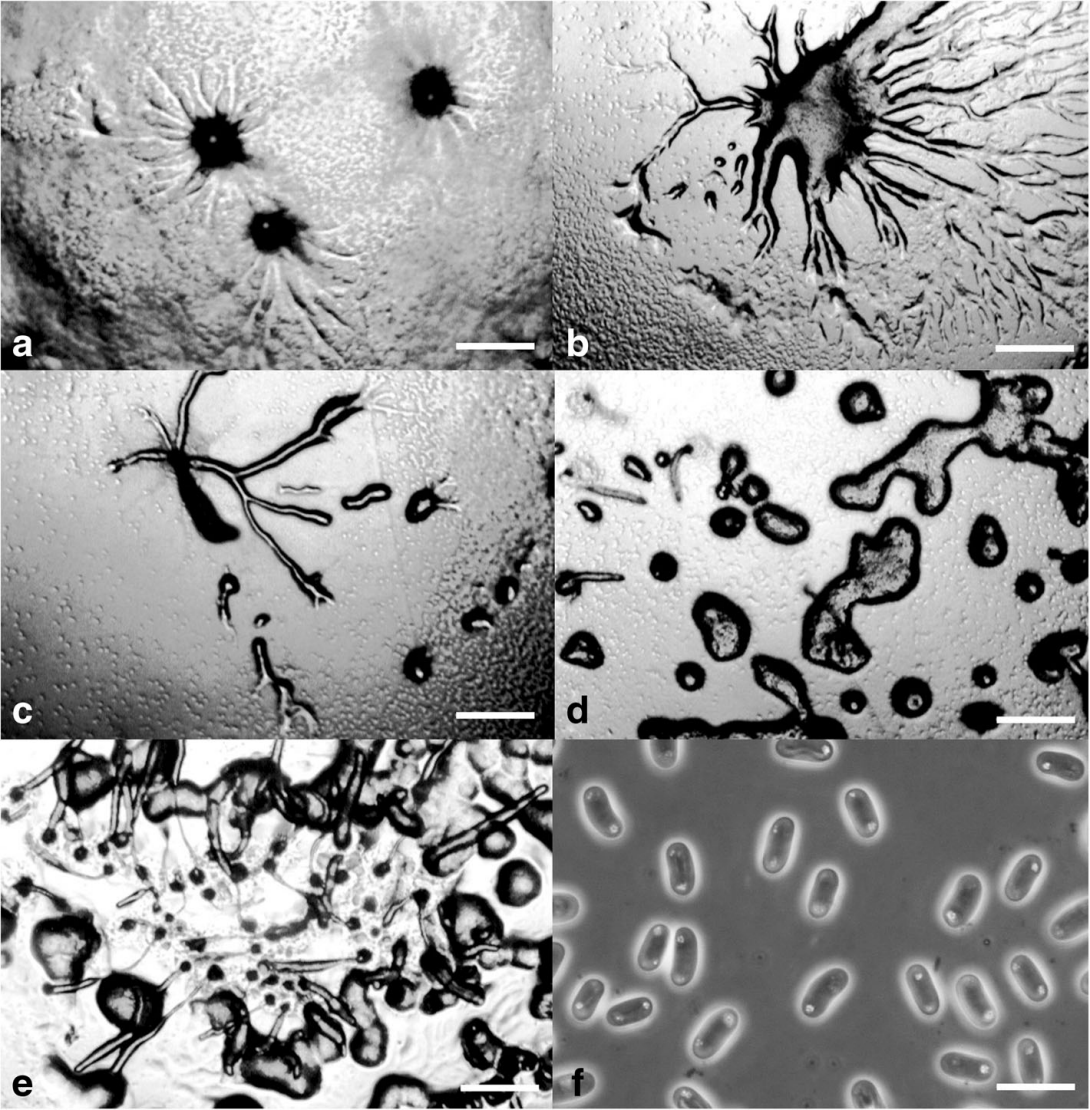
*Cavenderia bhumiboliana* (TH11CX). **a** Radial aggregations. **b** Large aggregation with a massive center and partitioning streams. **c** Late, subdividing aggregation with a rising sorogen. **d** Irregular, mounded aggregations. **e** Central cluster of sorogens that have arisen from a large aggregation surrounded by sorogens arising from smaller mounded aggregations. **f** Rather large, elliptical spores with consolidated polar granules. Scale bars: **a**–**e**: 225 μm; **f**: 10 μm

***Cavenderia bhumiboliana*** Vadell, Cavender, J.C. Landolt, A.L. Perrigo, P. Liu, and S.L. Stephenson, *sp. nov.* ***—*** Mycobank: MB827593; Figs. 4 and 5.

**Etymology.** This species is named in honor of the former king (Bhumibol Adulyadej) of Thailand.

**GenBank Accession Number.** HQ141523.

**Culture examined.** Thailand, Doi Inthanon National Park, tropical cloud forest, Chom Thong District, Chiang Mai Province, 18°35′32″N 98°29′12″E, isolated from a sample collected by Stephenson in January 2010, Landolt TH11CX (Holotype), Ex–Landolt TH11CX deposited in the Dicty Stock Center at Northwestern University (No. DBS0350789).

**Diagnosis. Sorocarps** solitary to clustered, prone, sinuous, decumbent, unbranched to branched, phototropic, mostly 1.5–4 mm in length, larger when aged, commonly 1.7–3.5(− 6) mm, in loose to tight clusters, crowded. **Sorophores** delicate, uneven, irregular, many times broken, with collapsed cells, mostly consisting of one tier of cells except for the lower section and bases, not tapered, sigmoid, with few branches in the upper section, 1–4, rarely more, the shortest at right angles, the larger ones at the low or middle point, ample, 500–700 μm long in old cultures, sorophores tapered from base to tips when within the sheath, 20–30 to 3–7 μm below the tip. Tips consisting of one cell, flexuous, sometimes terminating in an elongated cell (2–3 × 10 μm) or with an ample large flexuous cell with a piliform tip (range: 3–7 × 10–15 μm), or round, the tip may hold a globoid mass of slime matrix, with small tightly joined rather undifferentiated cells (15–25 μm diam.), sticky, then it is a compound flexuous capitate tip (Fig. 4e). Lower sorophores very irregular, with larger cells. Bases mostly clavate, curved or not, rounded one to two-celled when young (10–20 μm), becoming digitate and multicellular, 30–45 μm diam. Generally basal cells are very large, mostly protruding, globoid to acuminate, sometimes with adhered smaller cells forming a mass of stalk-cells below the base, all immersed in a dense yellowish matrix, highly granulated, and covered by a strong apron-like, granulated sheath (Fig. 4d). This end of the sorophore sheath is originally conical, becoming irregularly pleated and joined with the sticky slime. **Sori** globose to globoid, pale white to slightly creamy, mostly varying 30(20–100)–60(− 180) μm diam., apart from each other when in a cluster, drying out quickly when small. **Spores** elliptical-oblong, not perfectly regular in size nor in shape, broad, with prominent and large consolidated PG, polar to subpolar granules, sometimes dispersed and irregular in size and shape, many times surrounded by a clear halo, with other small dispersed granules and vacuoles within the heterogeneous content, not regular, (4.5–)6–7(− 8) x (2.5–)3.5–4(− 4.3) μm, 2.2 times longer than wide (Figs. 4f and 5f), sticky within the sorus. Most spores germinate immediately but some do not. **Myxamoebae** disperse widely at first, variable, 6.5–10 × 15 μm, with 2–4 median vacuoles, many remain unaggregated. **Aggregations** are irregular mounds with open tiny streams (Fig. 5a, d), when small (100–200 μm) sometimes gathering together or are streamed with anastomosed peripheral ends (300–500 μm). Streams become sharp, condensing into a few thick streams, then remain isolated as short sections or as blocks or round smaller masses usually surrounding a main irregular central mound, which gives rise to a loose cluster of early sorogens. **Sorogens** (Fig. 5e) in tight clusters, individual sorogens repel each other and may be of the same length or unequal. Early sorogens rise up singly or in pairs from the center of a pseudoplasmodium (Fig. 4a, b), short, club-shaped, conical, sometimes briefly migrating, then elongated and appearing as addressed tubes. Later larger developing sorogens migrate with stalk formation, with some late sorogens large (300–500 μm), sometimes massive, migrating with stalk formation, leaving open the extreme of the cone sheath or traces of it. **Sorocarps** stoloniferous (Fig. 4b, c) and small fruiting bodies are seen in situ after a time where sori have collapsed.

**Notes.** This species is very active and versatile, with fast and continuous growth and development in culture conditions for 2 months at 18–23 C and at a wide range of temperatures (10–15 C but up to 25–29 C). When the pseudoplasmodia condense into discrete sections, these may surround a main mound from which will rise up the first sorogen. After this condensation, the secondary smaller pseudoplasmodia will produce their early sorogens, producing a loose cluster with a main taller sorocarp two to four times larger than the companion ones (Fig. 4b). The central tallest body becomes decumbent and collapses on the agar. Some basal cells are unusually large, narrow, elongated and irregular. Yellow granulated mucilaginous matrix production is an important feature. Most spores germinate immediately but some do not. A group of features keeps this species separate from any other species that has been described. These are the loose clusters of very delicate, sigmoid and decumbent sorocarps of small to median size; the very irregular sorophores with digitate bases; spores that are very sticky, wide, with large subpolar to polar consolidated granules (below 2 μm), with halos and dispersed small granules in the spore body, of heterogeneous content; active myxamoebae and continuous development and growth for relatively long periods of time; the response to light exposure in which the slightly yellow color of pseudoplasmodia soon fades; migration of late sorogens that share a common origin and keep apart their cone-shaped terminal sheath before settling; enduring a wide span of temperatures and bacterial conditions (e.g., a mucoid strain of *E. coli*) without altering essential characters; and aggregations of two types, one small irregular unstreamed and streamed aggregations that become divided into thick enlarged, rounded early pseudoplasmodia. Some of these features resemble some characters of *C. aureostipes* (Cavender) S.Baldauf, S.Sheih & Thulin, but this species is smaller (mostly 1.5–4 mm in length), crowded, more delicate and has few or no branches.

**Fig. 6:**
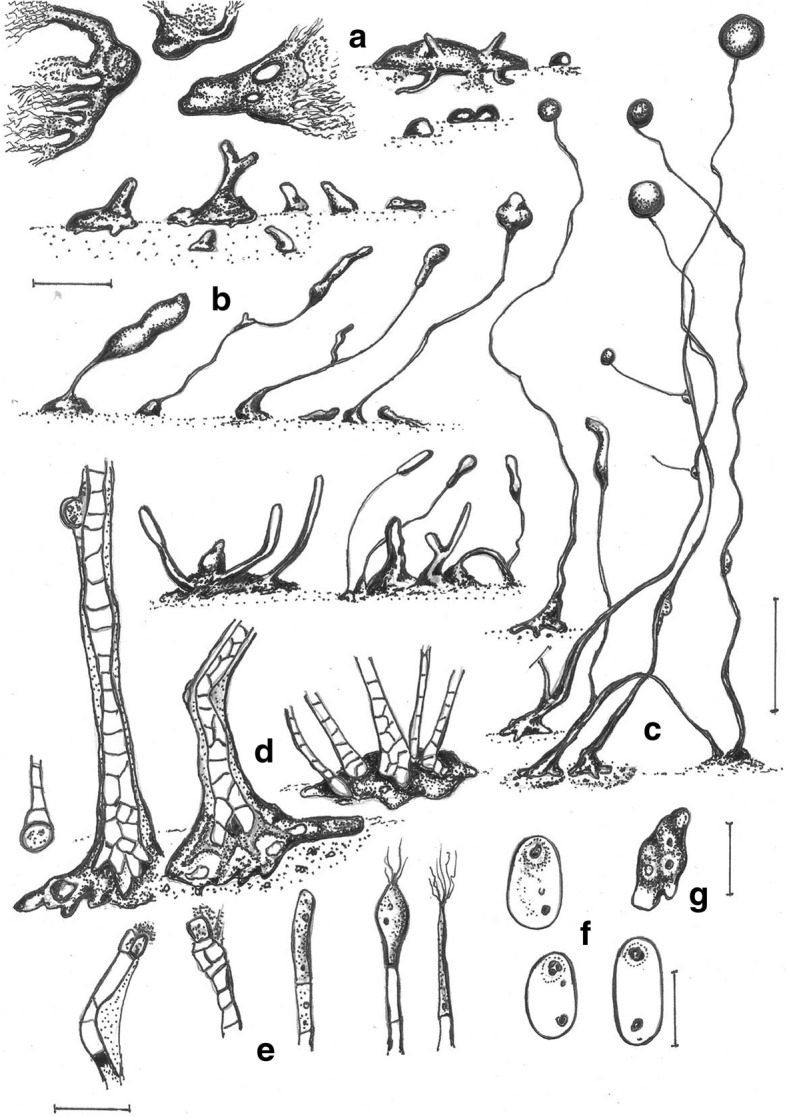
*Cavenderia protodigitata* (TH18BA). **a** Group of small to medium-sized aggregations (left, from above) and clustered late aggregation with short blocky streams (from the side, above, right) and small mounds (below, right). **b** A central main early sorogen surrounded by smaller settled sorogens (above), a series of development stages of solitary late sorogens from left to right, with migrating small sorogens (center), two clusters of early sorogens (below, left) and a loose cluster of sorogens at different stages of development (below, right). **c** Solitary unbranched sorocarp (above, left) and three sorocarps, two in a loose cluster and one with a stoloniferous habit (below, right), branches are scarce. **d** Bases, one round celled (left), two bases terminating in clavate to apron-like cells, one shows an irregular sorophore with a mass of dense granulated mucilage (center, left), the other shows a dense mucilage as a crampon below the base and within a lobulate slime with large dispersed cells (center, right), a tight cluster of lower sorophores (right). **e** Variable tips, one to two celled tips with dense slime (left), flexuous enlarged tip cell (center), two flexuous multipiliform tips, one is ampulla-shaped (right). **f** Elliptical slightly large spores with consolidated PG, one commonly large and with a halo. **g** Myxamoeba. Scale bars: **a**, **b**: 300 μm; **c**: 0.5 mm; **d**, **e**: 20 μm; **f**: 5 μm; **g**: 10 μm

**Fig. 7:**
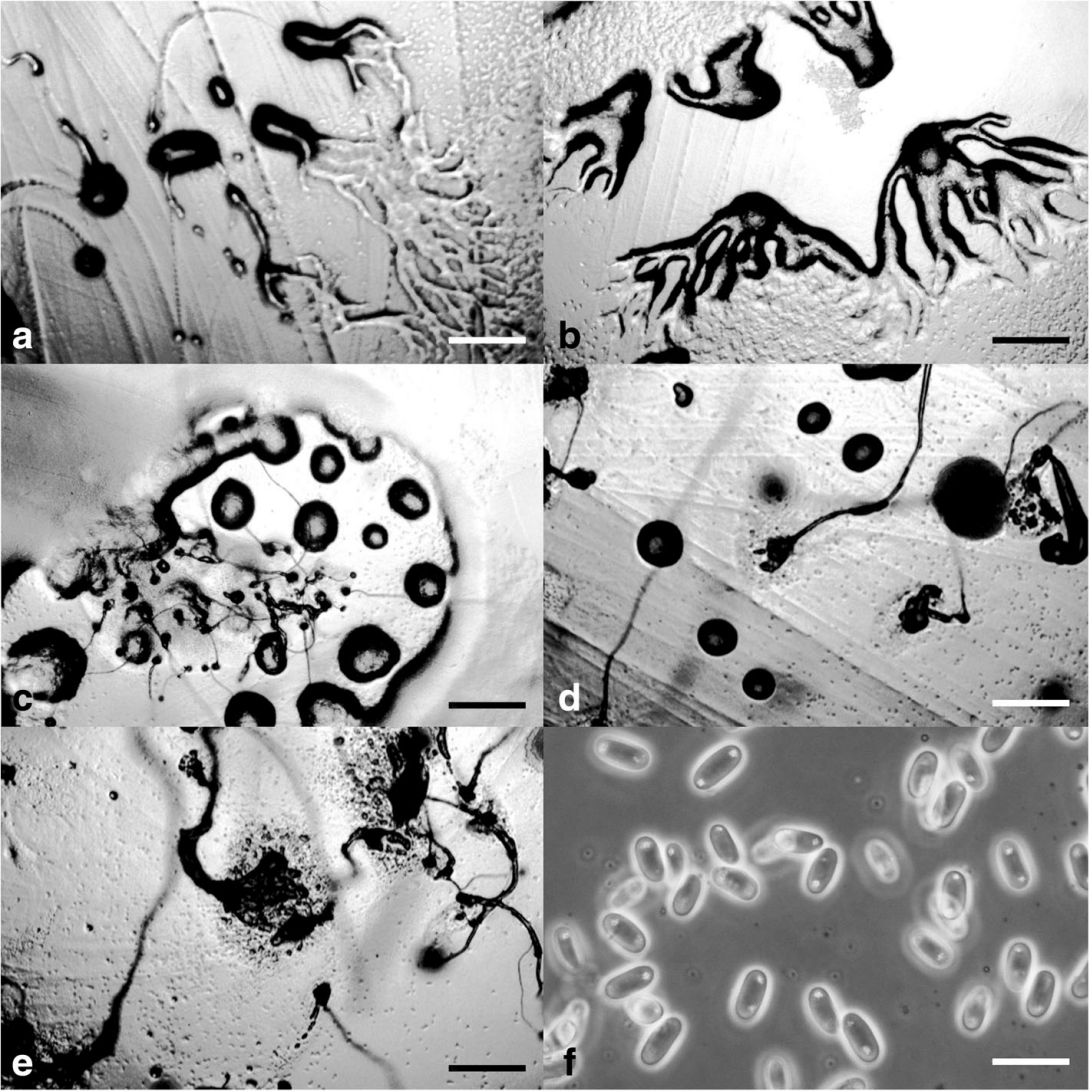
*Cavenderia protodigitata* (TH18BA). **a** Early aggregations with extensive streams. **b** Aggregations beginning to round up early. **c** Mounded aggregations surrounding early sorocarps that formed directly from germinated spores. **d, e** Fruiting body with a digitate base surrounded by small, mounded aggregations. **f**. Elliptical spores with polar granules. Scale bars: **a–e**: 225 μm; **f**: 10 μm

***Cavenderia protodigitata*** Vadell, Cavender, J.C. Landolt, A.L. Perrigo, P. Liu, and S.L. Stephenson, *sp. nov.* — MycoBank: MB827594; Figs. 6 and 7.

**Etymology.** The name refers to the presence of rudimentary digitations at the base.

**GenBank Accession Number.** MH745572.

**Culture examined.** Thailand: Doi Inthanon National Park, tropical cloud forest, Chom Thong District, Chiang Mai Province, 18°35′32″N 98°29′12″E, isolated from a sample collected by Stephenson in January 2010, Landolt TH18BA (Holotype), Ex–Landolt TH18BA deposited in the Dicty Stock Center at Northwestern University (No. DBS0350785).

**Diagnosis. Sorocarps** erect to prone, decumbent, solitary or in loose to tight clusters, commonly unbranched, although branches may develop from the lower sorophore as well as secondary smaller branches at the middle-upper point (100–300 μm), very sinuose, delicate and tortuous, stoloniferous 1–2 times, 0.8–3.5 mm long, with hyaline to white sorophores and sori but with yellowish pseudoplasmodia that fade very early during morphogenesis. Solitary sorocarps often surrounded by small pseudoplasmodial masses (Fig. 6b), by small earlier sorogens or by migrating sorogens. **Sorophores** are uneven, mostly consisting of one tier of cells, curved, cell sizes and shapes varying as well as sorophore diameter along the sorophore from 7 to 2 μm, except for the basal end, with a hyaline sheath of scattered granules and/or smaller, more abundant ones. Bases mostly clavate, made of 1–3 cells, (10–20 μm sect. Diam.), when one or two celled, the terminal cell is large, broad and clavate (3–5 × 7–8 μm) or simple-acuminate as an apron and smaller. If two celled, one is apron-like or protruding, irregular in shape. Large specimens may have the basal end with one acuminate, irregular thin cell or some short acuminate protruding cells (2–4), as short cellular aprons (shark tooth or cat claw-shaped), clavate, not expanded (Figs. 6d and 7d). Often large, unconnected cells at bases rather undifferentiated and located at the edges of the dense slime. Bases immersed into a very dense, sticky, granular slime and anchored by even more dense aprons of slime, non-granulated and distinctive. Bases in a tight cluster are united by the granular slime, within which some large stalk cells are spread (Fig. 6d). This slime is an essential part of the base. The sticky dense slime also is abundantly present at curves and branch bases as well as stationary guttae or drops alongside the sorocarp (10–20 μm). This condensed mucilaginous material has large irregularly angular granules of different sizes, extending up to the tip cell (not abundantly). Tips are variable, generally flexuous (as is sometimes true for the entire sorophore), often ending in one short to larger cell (2–3 × 4–7 μm), sometimes this terminal cell is curved or ends with some piliform filaments. Some tips are irregularly capitate with 2–4 cells of different sizes, mostly amorphous. **Sori** globose, hyaline to white, variable, 50–150 μm diam. **Spores** (Figs. 6f and 7f) elliptical-oblong, mostly regular in shape, (5–)6–7(− 8) x (2.5–)3–3.5(− 4) μm, germinating immediately, hyaline to vacuolated or with variable heterogeneous content, with two unequal medium to large consolidated, regular polar granules, and with other smaller dispersed granules, one of the two with an evident halo. **Myxamoebae** granulose and dense (10 × 6 μm), mostly small, migrating continuously to upper-sorophores, sorogens and pseudoplamodia, mostly limacine. Many myxamoebae remain migrating for a time. **Aggregations** are of a blocky type (Fig. 7a, b), irregular, surrounded generally by smaller pseudoplasmodial masses (100–300 μm) that approach the center or remain stationary. Larger **pseudoplasmodia** are irregular mounds from which tiny asynchronous sorogens arise. **Early sorogens** are very irregular in shape. Very early ones are club-shaped, becoming tube-like (50–70 μm), curved, granulose, branched or not, that separate from each other and become elongated, forming a small early-late sorogen with a tube-like elongated mass that may be hyaline or not (100–200 μm). **Late sorogens** become elongated rather rapidly and may anastomose with other masses. The formation of sori may be induced by water guttae or a drop at the terminus, with myxamoebae creeping into the droplets and positioning themselves.

**Notes.** Early aggregations (200 μm diam.) form from streams of myxamoebae but soon form elevated mounds. Streams are few (5–10) flat, thin, amply separated, entering the mound at right angles, many times interrupted and bifurcated, with amorphous ends. Some adjacent streams overlap without losing their individuality (each having a sheath). Streams are irregular to regular, not anastomosed at the ends, with some resemblance to the *C. aureostipes* type of aggregation. Pseudoplasmodia and sorogens have a yellow pigment that fades almost immediately. Myxamoebae enter into aggregations at any time and at any point. Optimum temperature for growth and development of this species is 22–25 C. The species tolerates 10–15 C, but is sensitive to lower temperatures as well as higher temperatures (29 C). In culture, myxamoebae disperse extensively within the bacteria streak and enter into the early streams of the aggregation, and pseudoplamodial masses form at any time and place, also climbing sorogens and sorocarps. For active observation, this species needs to be subcultured periodically, every two or 3 weeks, because it is ephemeral. This small to medium-sized tortuous, sinuose and delicate species of *Cavenderia* has aggregations and early pseudoplasmodia that resemble those of *C. aureostipes* in that masses of pseudoplasmodia are derived from the typical aggregation of that species and consolidated polar granules are similar to that species. However, the solitary and clustered habits, differential length, kind of sorophore, tips and bases distinguish this species from any other *C. aureostipes*-like strains or any other similar species. Spores are also distinctive as well as the rates at which different sorogens develop. This new species differs from *C. aureostipes* in its smaller size, clearly distinguishable and very delicate sorocarps with a crampon-like bases, the almost always unbranched sorophores, smaller differential aggregations and non-pigmented sorocarps, This strain is larger than TH18 (another isolate from Thailand) and its sorocarps are more regular, more sigmoid and broken. Tips and bases vary and are different compared to small similar species, and bases have rudimentary cellular aprons distinctive from other species.

**Fig. 8:**
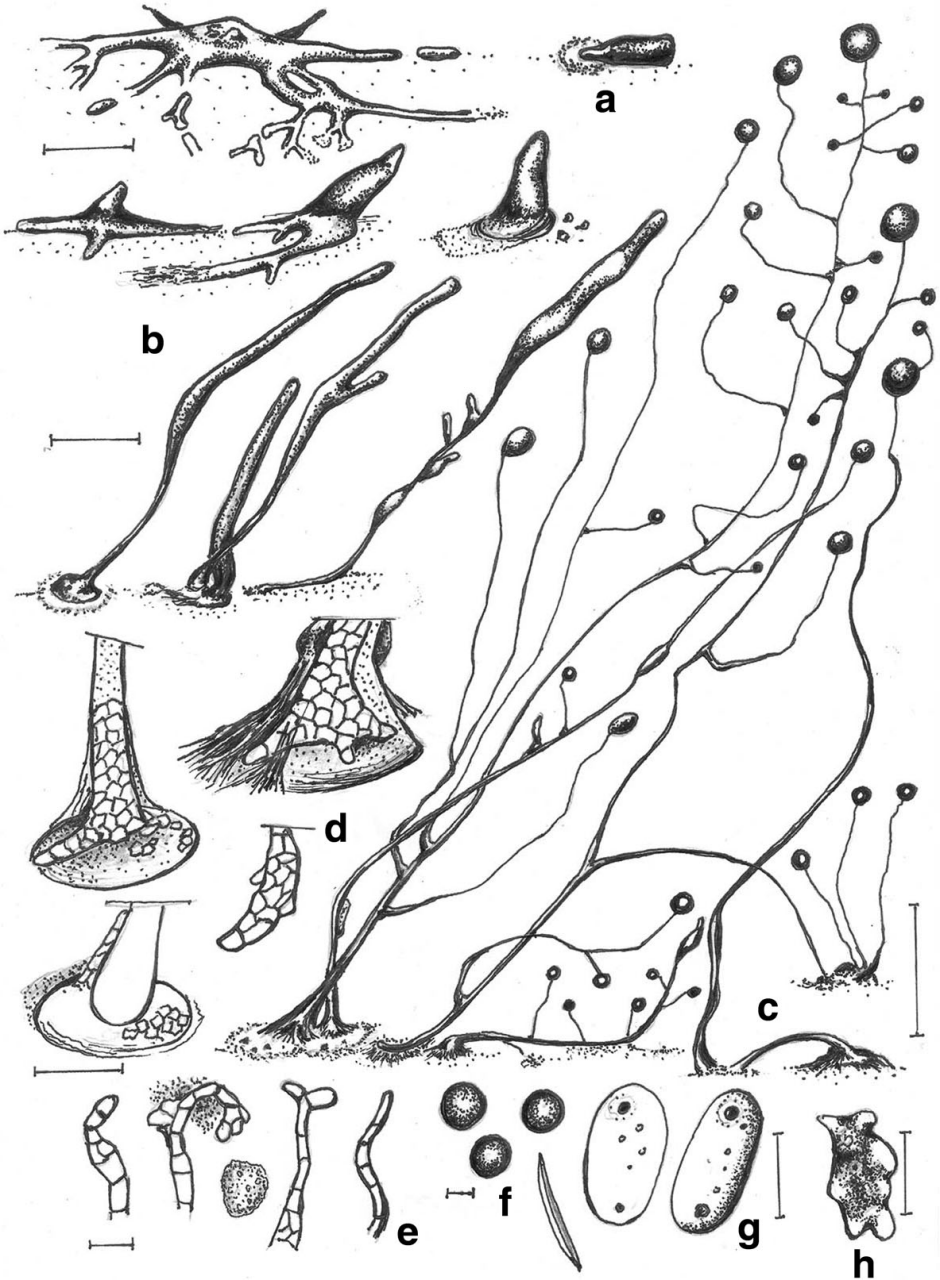
*Cavenderia pseudoaureostipes* (TH39A). **a** Irregular *violaceum*-type aggregation, crab-like shape (left), a migrating small pseudoplasmodium, which ends as a settled mound (right). **b** Early central solitary sorogens with radiate ample streams (above, left), migrating with rest of streams (above, center), migrating without streams (above, right), late solitary unbranched sorogen (below left), clustered sorogens (below, center), branched late sorogen with prostrate lower part (below right). **c** Highly branched tightly clustered fruiting bodies (left), loose clustered prostrate and refruiting sorocarps (center), a smaller solitary unbranched stoloniferous sorocarp refruiting from a sorus (right). **d** Digitate bases on a disk of slime surrounded by smaller polygonal cells, the sheaths are densely fascicular (above, left and right), out of scale, scheme of a rounded base on a disk with basal cells (below, left) and curved clavate base with protruding cells (below, right). **e** Curved tips simple to capitate (left), a mass of dense mucilage close to the tip (center), capitate and simple tip (right). **f** Microcysts. **g** Elliptical regular spores with prominent consolidated PG and small vacuoles, a folded thin half of a spore-coat (left). **h** Myxamoeba. Scale bars: **a**: 300 μm; **b**: 200 μm; **c**: 0.5 mm; **d**: 25 μm; **e**: 10 μm; **f**: 2 μm; **g**: 5 μm; **h**: 10 μm

**Fig. 9:**
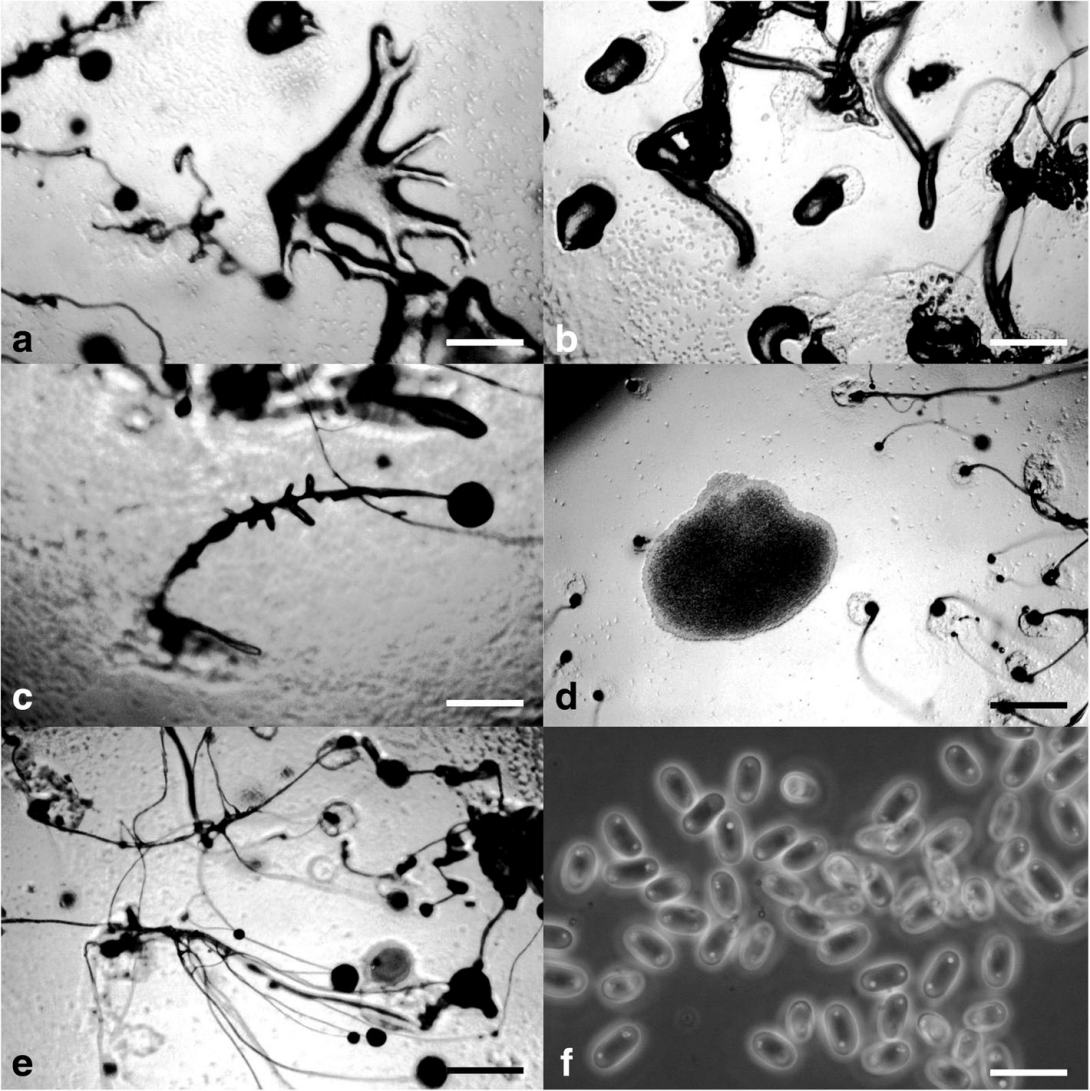
*Cavenderia pseudoaureostipes* (TH39A). **a** Older aggregation with a large center. **b** Developing single pseudoplasmodia. **c**. Developing many branched sorocarp. **d** Sorocarps with expanded bases in slime envelopes. Ungerminated spore mass. **e** Branched soprocarp. **f** Elliptical spores with consolidated polar granules. Scale bars: **a**–**e**: 225 μm; **f**: 10 μm

***Cavenderia pseudoaureostipes*** Vadell, Cavender, J.C. Landolt, A.L. Perrigo, P. Liu, and S.L. Stephenson, *sp. nov.* — MycoBank: MB827595; Figs. 8 and 9.

**Etymology.** The name refers to the similarity of this species to *C. aureostipes*.

**GenBank Accession Number.** HQ141518.

**Culture examined.** Thailand: Wat Pong Ao (Temple) Just south of Mae Chan, evergreen tropical forest, Mae Chan District, Chiang Rai Province, 20°08′48.3″N 99°51′09.2″E, isolated from a sample collected by Stephenson in January 2010, Landolt TH39A (Holotype), EX–Landolt TH39A deposited in the Dicty Stock Center at Northwestern University (No. DBS0350781).

**Diagnosis. Sorocarps** erect, prone to decumbent, solitary to loosely clustered, stoloniferous, 2–5 mm long, slender, branched mostly at the midpoint and in the upper section. **Sorophore** straight to curved, yellow, consisting of one tier of cells except for lower section, which consists of 2–4 cells tapering from nearly 40 to 5 μm at the subtip, cells at the middle section are regularly polyhedric. Bases rounded to irregularly clavate, elongated, curved or not, 2–12 celled, with protruding cells, variable when aged and becoming massive (10–90 μm), or with crampons (protruding larger cells), immersed within an irregular, large discoid cushion of dense slime and small cells (40–200 μm diam.), surrounded irregularly by a halo of slime and dispersed cells (Fig. 8d). Concentric lines of a hyaline matrix extend outward on the flattened thin edge of the discoid basal formation. Tips irregularly capitate, mostly hammer-like and curved (10–20 μm) or ending in one larger club-shaped cell (Fig. 8e). Branches are small to large, numerous (100–700 μm in length), they come from independent pseudoplasmodial ascending masses (Figs. 8b and 9c). Sometimes elongated medial branches are monochasial. **Sori** globose, white, (50–)80–200(− 250) μm diam., may refruit. **Spores** are elliptical-oblong, very regular in shape, wide, (6–)6.5–8.5 x (3–)3.8(− 4.5) μm (ave. 7.0 × 3.5 μm), mostly with consolidated polar granules, many of these rounded, surrounded by clear halos, sometimes subpolar and/or with dispersed consolidated smaller granules along with many tiny vacuoles (Figs. 8g and 9f). Spores germinate immediately. **Aggregations** (Figs. 8a and 9a) are radial, crab-like (500–600 μm) or with few extended streams (generally two), that ultimately become partite. Streams may be open ended or not. Small masses migrate short distances or extensively when the early sorogen is formed, followed by a long, pointed pseudoplasmodial mass (1–3 pointed bodies). **Early sorogens** arise from large yellow pseudoplasmodia (0.7–1.0 mm), emerging singly from the center or in loose clusters. Pseudoplasmodia also may be mounded with sharply delineated edges. **Late sorogens** very elongated, active, stoloniferous and migrating (Fig. 8b). **Microcysts** present (Fig. 8f). Myxamoebae large, yellow, 5–8 × 10–15 μm, with many vacuoles and granules (Fig. 8h).

**Notes.** This yellow species is very active when cultivated at 22–26 C on *E. coli*, capable of continuous growth and development even at higher temperatures (25–29 C) and retaining its color for a long time, but may not survive low temperatures (development stops at 10 C). The sorophore morphology is quite variable, although most of the cells keep a regular shape. The slime sheath is densely granular. Some early unbranched sorocarps migrate 200–300 μm as they continue development. The sori contain a dense matrix of slime that make them sticky. Collapsed sori refruit immediately in situ, in old cultures the smaller sori dry out quickly, while the large ones are resistant to low humidity. Mature luxurious cultures may show coremiform sorocarps. Some branches may also become bifurcate. Masses of dense granulated slime add support to the lower sorophores. Irregular and strong aprons of dense slime sheaths keep the bases firmly attached to the substrate. This species tends to confine itself to the culture bacterial streak. The spore coat is very thin, splits longitudinally and rolls up, soon appearing as a line under magnification. This species, as well as *Cavenderia subdiscoidea* (TH1A), may have coevolved with *C. aureostipes*, considering their similar morphologies.

**Fig. 10:**
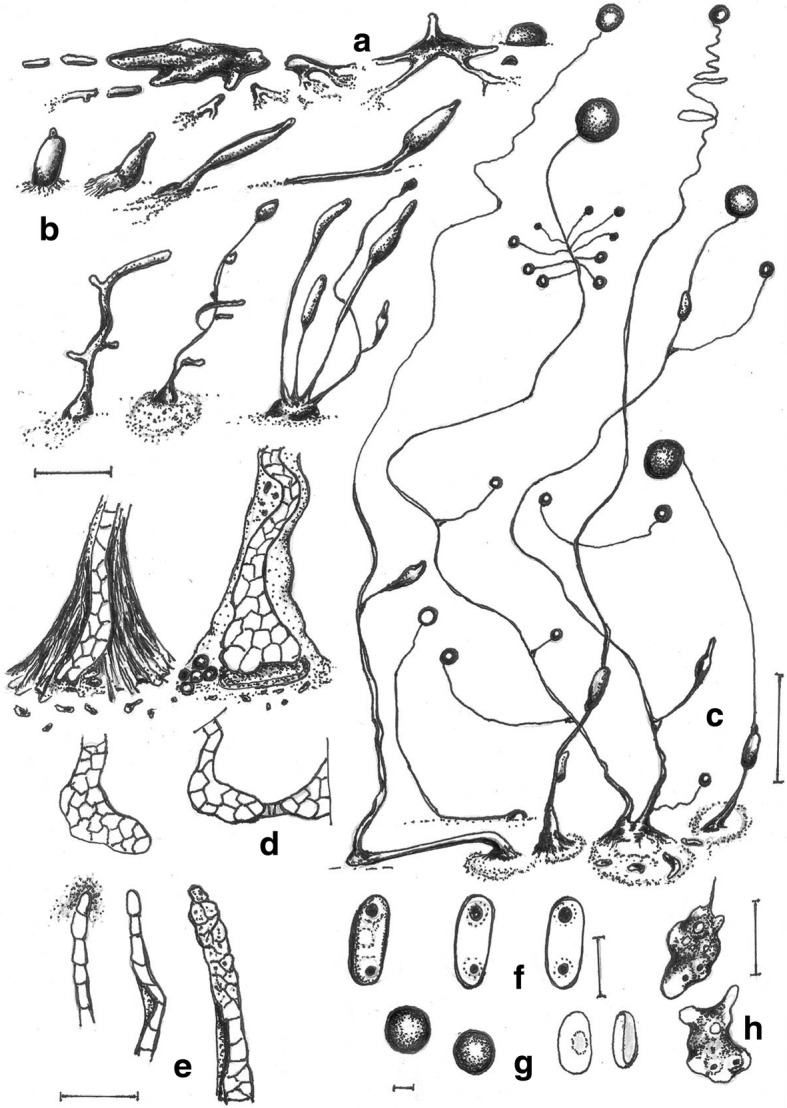
*Cavenderia subdiscoidea* (TH1A). **a**. Late median *violaceum*-type aggregation (left) and a small mound, sometimes with radiate streams (right) or with minute ample streams (above). **b**. A series of developing early solitary sorogens, club-shaped, pyriform, then elongated and migrating free when small (above left) and with a stalk formation (above right). A solitary branched early-late sorogen (below, left and center) and a cluster of late sorogens (below, right). **c**. Three solitary sorocarps, two of them with prostrate lower sorophores of different sizes (left), a cluster of two large sorocarps, with some branches and with ascending masses of pseudoplasmodia and patchy areas of slime and myxamoebae at the base, one sorocarp with a very elongated and weaving tip (center), a smaller curved unbranched solitary fruiting body (right). A halo surrounding bases is represented in all figures. **d** Two bases, a clavate slightly digitate base strongly attached by aprons of sheath (above, left), an irregular round base on a disk of dense slime, cells as microcysts immersed in a dense slime (above, right). Two massive curved clavate bases, one with a cell connection with another within a cluster (below). **e** Three tips, simple celled with abundant slime (left), a terminal broken segment (center) a flexuous capitate tip (right), all cells of regular to small size. **f**. Narrow large elliptical spores with prominent large regular consolidated PG with halos. Below, two spore coats (out of scale). **g** Microcysts. **h** Active myxamoebae with two-four large-median vacuoles, two main black spots. Scale bars: **a**, **b**: 300 μm; **c**: 0.5 mm; **d**, **e**: 15 μm; **f**: 6 μm; **g**: 2 μm; **h**: 10 μm

**Fig. 11:**
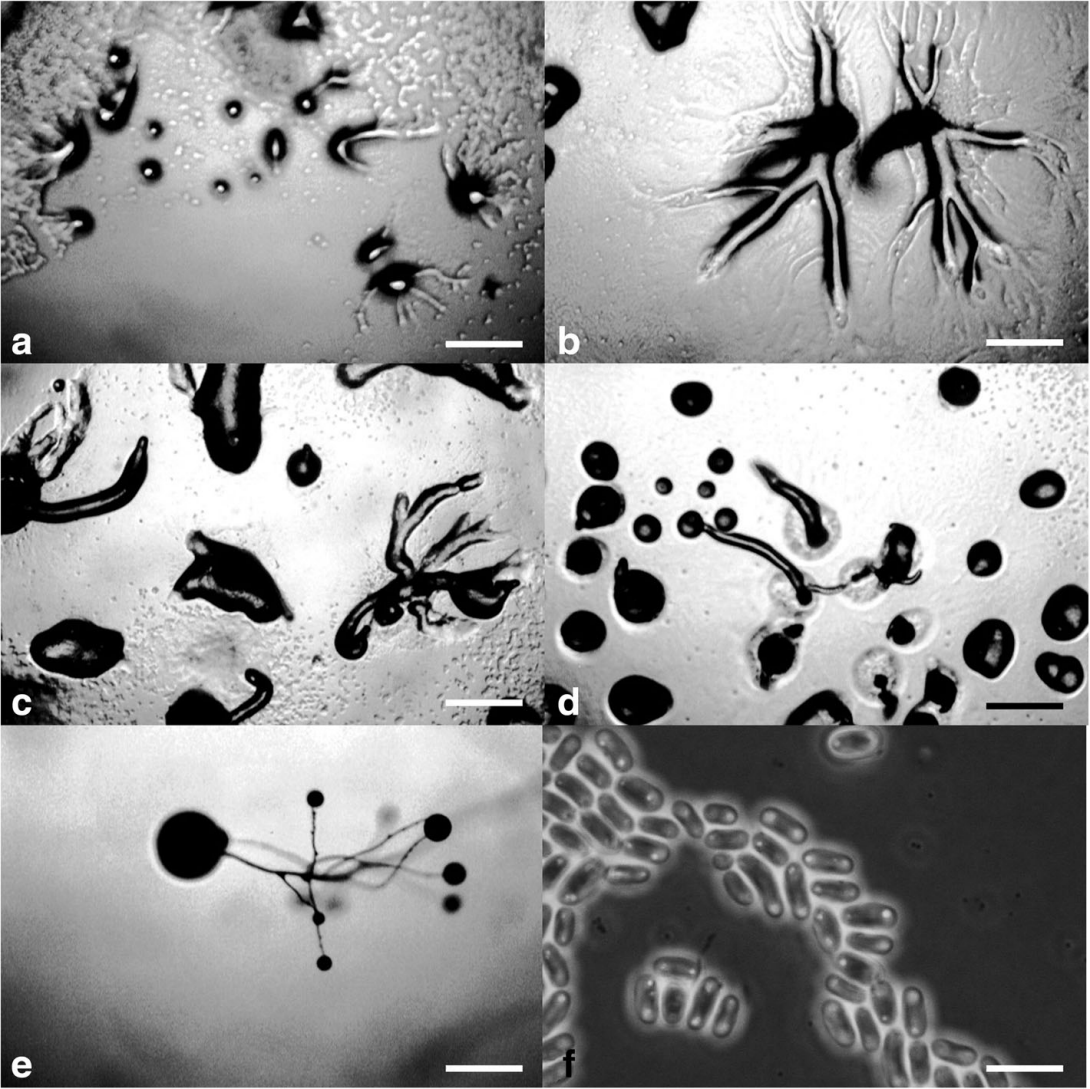
*Cavenderia subdiscoidea* (TH1A). **a** Early small aggregations, both mounded and with radial streams. **b** Later aggregations with blunt, blocky streams and rising sorogens. **c** Late aggregations with single sorogens and with persistent streams, some mounded aggregations. **d** Mounded aggregations producing single sorogens having conspicuous slime bases. **e** Typical branched sorocarp. **f** Large elliptical spores with consolidated polar granules. Scale bars: **a–e**: 225 μm; **f**: 10 μm

***Cavenderia subdiscoidea*** Vadell, Cavender, J.C. Landolt, A.L. Perrigo, P. Liu, and S.L. Stephenson, *sp. nov.* — MycoBank: MB827596; Figs. 10 and 11.

**Etymology.** The name refers to the typical basal slime disk.

**GenBank Accession Number.** HQ141515.

**Culture examined.** Thailand: Mushroom Research Center, montane tropical forest, 128 Moo3, Bahn Pa Dheng, T. Pa Pae, A. Mae Taeng, Chiang Mai 50,150, 19° 07.200’ N, 98° 44.044′ E, isolated from a sample collected by Stephenson in January 2010, Landolt TH1A (Holotype), Ex–Landolt TH1A deposited in the Dicty Stock Center at Northwestern University (No. DBS0350779).

**Diagnosis. Sorocarps** solitary or in tight to loose clusters, erect to prone, sigmoid, decumbent, unbranched to branched, small to medium 1–5 to 7 mm in length, commonly 1.5–3 mm high. Smaller branches in solitary sorocarps are sometimes bifid and/or the sorocarps have a few small to large branches. **Sorophores** slender, then curved many times, mostly with the lower part prostrate and forming a stalk thereafter, with short crowded branches in the upper section (1–10) and a few large branches in the lower-middle sorophores (Fig. 10c), inserted in right angles (0.5–1 mm in length), slightly and irregularly tapered, lower sorophores with nodes of more cells per section (4–6 cells) with granular mucilaginous material and with a drastic curve close to the base, the middle-upper sorophore more regular, although with alternate sections of one to two tiers of cells. Branches sometimes coincident (2, 3 or more). Tips one celled, flexuous or not, 5–7 μm diam., generally the terminal segment is flexuous as two lines of small cells, with large granules inside (range of 100–150 μm long), sticky. Lower sorophores very irregular, with larger cells. Base round, lobed to clavate multicellular, 30–45 μm diam., on an irregular basal disk of slime, or smaller (one celled). Sometimes surrounded by a halo of tiny pseudoplasmodia and slime. Base with a plane end or with an enlarged tubular clavate cell, when joined in a tight cluster, sometimes with two bases in contact with their bases sharing the same dense abundant basal granulose matrix of slime and the granular sheath, also sharing a short bridge of stalk cells that connect them (Fig. 10d). These bases support diverging sorophores (when older). **Sori** globose, white to hyaline, 50–90(− 200) μm diam., apart from each other in a cluster, although many times tangled and collapsing when decumbent, then they refruit. **Spores** (Figs. 10f and 11f) elliptical-oblong, regular, slightly narrow, 6–8 × 2.8–3.8 μm (ave. 7.0 × 3.3 μm), with consolidated, compacted polar granules, dense, round, with clear halos during dormancy. Spores soon enlarge when in contact with the humid substrate and the spore body becomes heterogeneous and granules enlarge. Most spores germinate immediately (21–24 C). **Myxamoebae** active, spores germinate by dissolving part of the delicate spore-coat, which remains uncollapsed, transparent (Fig. 10h). Condensed old polar granules migrate later into the cytoplasm of the myxamoebae, which then develops an active internal movement, showing in sequence 1–4 large to median vacuoles. **Aggregations** are irregular mounds at first (Fig. 11a), then developing short ample streams. These flat, sometimes anastomosed, streams becoming large (0.6–1.5 mm). Streams become disrupted and form blocky sharp streams (Fig. 11b, c) and remain as short enlarged masses or smaller round ones (like what is typical of *Polysphondylium violaceum*). **Pseudoplasmodia** yellow at first, fading with light and age. **Early sorogens** (Fig. 11e) rise up from the center of a pseudoplasmodium, migrate briefly, club-shaped, sometimes branched and soon collapsing. **Late sorogens** elongated, irregular and curved, migrate with stalk formation (Fig. 10b). **Sorocarps** (Fig. 11e) stoloniferous and development of sorocarps begins anew immediately. **Microcysts** present.

**Notes.** This species is very active when cultivated at 21–24 C on *E. coli*, with continuous fast growth and development (until reaching the lid of the culture dish), at low temperatures (10–15 C) as well as high temperatures (25–29 C). Masses of pseudoplasmodia continuously creep up the sorocarps. Feeding myxamoebae clear an expanding zone when on diluted *E. coli*, leaving at the center of the cleared zone a few ungerminated spores. Large myxamoebae remain on the substrate unaggregated but eventually adhere to other masses or stalks. A granulated mucilaginous matrix is important for the support of bases and branches. The sheath is sticky and not well defined, The upper sorophore may remain flexuous). Some spores do not germinate immediately. The inner matrix of the sorus appears to inhibit the germination of spores. A group of features separates this species from any other described dictyostelids [e.g.: *C. aureostipes, C. medusoides, C. aureum* Olive and/or any other species with yellow, branched/unbranched sorocarps, PG (+) spores, and the “*violaceum*” type of aggregation]. These are the loose clusters of sigmoid and decumbent sorocarps of small to medium size; two-three sorocarps sharing by their bases a common basal slime matrix; irregular sorophores with large granules; spores sticky, wide, with polar consolidated granules, with halos and clear areas of the spore body, soon becoming heterogeneous; active myxamoebae and continuous development and growth; somewhat phototropic; yellow pseudoplasmodia that soon fade; commonly tightly clustered bases that share a large slime matrix apron; and aggregations are mound-like that join together, as well as those with large radiating streams that subdivide as in *C. aureostipes*. Some of these features resemble characters of *C. aureostipes*. Although this species is crowded, with fewer large branches, it somewhat resembles strain TH19B (another isolate from Thailand) and a strain (DHI, yet to be described) from Ohio but is smaller. As is the case for the other species described herein, *C. subdiscoidea* may endure the conditions present in a dry microenvironmental culture (xeric media) for more than a month and the sorocarps remain viable after 1 year within a range of temperatures from 5C to − 18 C.

## Discussion

In the survey of dictyostelids of Southeast Asia carried out in 1970 by Cavender, samples were collected from three localities in Thailand [8]. These were Kao Yai National Park in southern Thailand along with collection sites near Chiang Dao and Chiang Mai in northern Thailand. All three sites were characterized by tropical semi-deciduous forests, and the underlying bedrock for northern Thailand is limestone. The data from this survey were summarized by Cavender [8]. Nine species were isolated from samples collected in Thailand, including a new species in the genus *Cavenderia* (*C. bifurcata*) from Chiang Dao. This species was also isolated from Indonesia along with another species—*C. multistipes*. However, the more common and widely distributed *C. aureostipes* was not isolated from Thailand or from any of the other countries of Southeast Asia included in the survey. An unidentified branched form with a yellow stalk was isolated at Chiang Mai in northern Thailand. Based on the morphology, this may have been what we have described as *C. pseudoaureostipes* in this paper, although it is not possible to confirm this. Chiang Dao and Chiang Mai also yielded an unidentified dictyostelid with polar granules in the spores (PG+). Based on this character, this was probably a member of the genus *Raperostelium*, perhaps similar to *R. monochasioides* (H.Hagiw.) S. Baldauf, S. Sheikh & Thulin or possibly an undescribed species. In terms of overall frequency, density and percent presence, the small PG+ dictyostelids ranked fifth in Southeast Asia. *Dictyostelium purpureum, D. mucoroides, Heterostelium pallidum*, and *Polysphondylium violaceum* were the most common, as is also the case for other countries in Southeast Asia [4]. The *H. pallidum* isolate recovered in the earlier survey was probably a mixture of species that have not yet been described. Other species recorded were *Coremiostelium polycephalum* and three crampon-based species—*Hagiwaraea rhizopodium* (Raper & Fennell) S.Baldauf, S.Sheikh & Thulin, *H. lavandula* and *H. vinaceofusca* (Raper & Fennell) S.Baldauf, S.Sheikh & Thulin. The latter had a high frequency and density at Chiang Dao, while *H. rhizopodium* had a high frequency in Kao Yai National Park. Diversities of the assemblages of species present in Thailand were similar to those found in other parts of Southeast Asia, except for Udjong Kulon in Indonesia, which had a greater diversity [8].

The soil samples examined in the present study were obtained from a tropical broadleaf forest at the Wat Pong Ao Temple (a study site near Chiang Rai), from a montane broadleaf forest at the Mushroom Research Center near Chiang Mai, and from a tropical cloud forest at ca. 2.500 m elevation in Doi Inthanon National Park. In addition, as already noted, species of dictyostelids isolated following the initial study by Cavender included *Acytostelium* sp. (probably *A. subglobosum*), *Dictyostelium giganteum*, *Tieghemostelium lacteum*, *D. sphaeocephalum*, *Heterostelium candidum* and a group of unidentified isolates [12]. Five of the latter were found to represent species new to science and are described herein. SSU sequencing of two isolates from Doi Inthanon cloud forest by Perrigo [13] has shown that they are closely related to each other and were placed in their own clade (part of the *C. aureostipes* complex) along with *C. myxobasis,* a species known only from canopy epiphyte soil collected in Queensland, Australia [14]. The two Doi Inthanon tropical cloud forest species (*C. bhumiboliana* and *C. protodigitata*) probably evolved in situ did not occur in tropical lowland forest but were found only at higher elevations. Two other species (*C. aureostabilis* and *C. subdiscoidea*) were recorded from montane forests. The single species (*C. pseudoaureostipes*) described from the tropical forest at Chiang Rai is the largest species isolated in the present study and also the one most similar to the commonly isolated *C. aureostipes* [15]. All of the isolates share a number of morphological similarities (Table 1) and differ mostly in size and complexity of their fruiting bodies. For example, all have branching sorocarps, which are more complex with a larger number of branches in the larger species. All have clustered sorocarps to some extent, and all have similar PG+ spores which vary somewhat in size and shape among the various species. All tend to produce a yellow pigmentation that is more persistent in the larger species. All may produce mounded and/or streaming aggregations that become larger and more streamed with sorocarp size. The streams break up during aggregation, forming additional centers and sorocarps. This type of aggregation is referred to as the *Polysphondylium violaceum* type [3].

Other features described herein for these new species are the delicacy of the sorophores, the extent of migration, abundance of slime, and temperature tolerance. All of these are greater for the smaller species, except for migration. The smaller species were found only in the tropical cloud forest, and the larger species were recovered at lower elevations. The apparent evidence of speciation observed may have originated from an initial colonization of a *C. aureostipes* type ancestor such as *C. pseudoaureostipes*, associated with a tropical forest but which may have given rise to a series of well-adapted smaller types in higher elevation forests. The latter forests provide a more acidic substrate (soils derived from granite bedrock) and the soils also have higher levels of organic matter. The reduction in size, increase in clustering and more abundant slime at the bases are probably adaptations to the cooler, more acidic microenvironment. The tendency to form mounds during aggregation and the production of single sorocarps from the aggregations have been observed for low concentration bacterial plates, and it is hypothesized that this represents an adaptation to the low densities of bacteria which exist at the ca. 2.500 m elevation of the study site in the Doi Inthanon tropical cloud forest.

Characters that appear to be correlated with the small size are the short migration, adhesiveness of the mostly basal slime, a low degree of branching, and the frequent formation of small mounds. It is likely that these species are well adapted to short distance dispersal within the litter-soil microenvironment, which is in agreement with the close phylogenetic relationships shown in the sequencing. The two new smaller species found at high elevations on Doi Inthamon commonly produced sorophores with flexuous ends and bases with a well-defined, acutely clavate shape or with protruding sharp cells resembling short digitations. These may be cases of a gradual disappearance of a major character (e.g., a digitate base). The closest relative both morphologically (Table 1) and genetically is *C. myxobasis* [14]. This species was found in canopy soil, which shares a high level of organic matter characteristic of the microenvironment in which the Doi Inthanon cloud forest species occur. Some morphological features (e.g., the yellow pigmentation) are also shared with *C. medusoides* (from the tropical forest of Tikal in Central America [16] and an unidentified species of *Cavenderia* (Thailand clade of the *C. aureostipes* complex) reported from a tropical forest in Thailand by Perrigo [13] as well as one of the species (*C. pseudoaureostipes*) reported herein.

Sequencing has shown that these cloud forest species are closely related and most probably evolved in situ. As already noted, the species composition and diversity of the assemblage of dictyostelids in Thailand are generally similar to what has been reported in previous studies of both the dictyostelids of Southeast Asia [8] as well as the American tropics. In both regions, those species that appear to be endemic are rare species. The endemic rare species appear in this case to be surviving as organisms adapted to a cool environment characterized by high levels of organic matter. In fact, as a clue for this observation, survivorship of these species in the laboratory appears related to the abundance of the slime matrix at the bases and tips of their sorocarps, which enhance these slimy small to median species to survive long periods of dry microenvironmental conditions (more than 1 month in xeric media) as well as low temperatures (from 5 C to − 18 C) for longer periods (more than 8 months), that are similar to the overall climatic conditions which exist in highland and lowland forest environments at these latitudes.

## Conclusions

Reported herein are the results of a survey for dictyostelids carried out in northern Thailand. Our discovery of 15 taxa of dictyostelids, including five new species (*Cavenderia aureostabilis*, *C*. *bhumiboliana*, *C*. *protodigitata*, *C*. *pseudoaureostipes*, and *C*. *subdiscoidea*), expands what is known about the biogeography and ecology of dictyostelids. The results of this survey confirm both the relatively high diversity of dictyostelids in the general study area and the presence of an assemblage of species similar to those found in other parts of Southeast Asia. The primary exception of this pattern, based on available data, is Udjong Kulon in Indonesia, which was characterized by a greater diversity [8]. In addition, the present study provides evidence of a locality where relatively rapid evolution and speciation in dictyostelids appears to occur in an environment where it might not be expected, based on previous studies carried out in the tropics. For example, Cavender et al. [17] observed that “considerable evolution had occurred in the small dictyostelids, especially in the development of streams.” Stream formation is believed to be a significant evolutionary advance for dictyostelids, and this appeared to be the case with the small highland species described herein. The relatively limited microenvironmental dispersion of small species of dictyostelids seems to be related to a dense slime layer that surrounds the base of the sorophore, which was the case for some species of Australian dictyostelids (e.g., the cited *C. myxobasis*) [12] as well as some of the new species from Argentina “that produce sori with dense slime that limited spore dispersal” [18]. Presumably, the limited dispersal of spores forces a particular strain to become highly adapted to a restricted area over long periods of time, a situation which might be expected to enhance speciation.

Morphological characters *per se* still represent an exceedingly useful diagnostic method to discriminate different new species of dictyostelids [19]. Clearly, a phylogenetic approach based upon molecular biology reinforces the recognition of new species, but this does not lessen the value of traditional macroscopic observations of characters, habits and adaptations when studying the spatial distribution of dictyostelids in old and new environments.

## Methods

### Sampling, isolation and cultivation

Forty soil samples were collected from four localities in northern Thailand. Ten samples consisting of 10–20 g from the soil/humus layer were collected at each locality and placed in sterile bags until they could be cultured. Isolation procedures followed Cavender and Raper [5] and are briefly summarized here. Each sample was weighed and distilled water was added to obtain a final 1:25 dilution of sample material. Aliquots of 0.5 mL of this suspension were added to each of two or three 95 × 15 mm culture plates prepared with hay (leached and dried, primarily composed of *Poa* sp.) infusion agar [2]. This produced a final dilution of 0.02 g of soil per plate. Approximately 0.4 mL of a heavy suspension of *E. coli* was added to each culture plate, and plates were incubated under diffuse light at 20–25 C. Each plate was examined at least once a day for several days following the appearance of initial aggregations, and the location of each aggregate clone marked. When necessary, isolates were subcultured to facilitate identification. Taxonomic nomenclature used herein follows Sheikh et al. [1].

### Observations of morphological features

The five isolates that were not morphologically consistent with any known species were further investigated according to their morphology and molecular characteristics. Subcultured clones of these isolates were studied in parallel but independently by Vadell along with Cavender and Landolt. Cultures in the Vadell laboratory were observed with a 50–200X lens, either in hydric conditions or in xeric media conditions to observe the effects of dehydration and conservation of the hydric contents of the sori over time, migration, stolon formation and sorogen development. In addition, aliquots of 200 μmL of whole cultures (sorocarps and previous developmental stages present in a particular culture) of each strain were kept frozen in 500 μmL eppendorf tubes at − 18 C for 1 year. Observations of early aggregations, pseudoplasmodia, and fruiting bodies were made after 2–30 d incubation under diffuse illumination at 18–26 C as described in a number of previous studies [19, 20]. The general taxonomy criteria used for the various morphological features observed were based on Raper [2].

A morphological description of each of the five new species is presented herein, and a comparison of key diagnostic morphological characteristics is given in Table 1. Cultures are freely available from the Dicty Stock Center (dictybase.org) at Northwestern University (Evanston, IL, USA).

### DNA isolation, PCR amplification, sequencing and phylogenetic analysis

The ribosomal small subunit (SSU) of all five new species was sequenced for phylogenetic analyses. DNA was extracted from pure isolates using the technique described in Perrigo [13] and Polymerase chain reaction (PCR) amplification of the SSU was carried out using the primers described by Medlin et al. [21] and Schaap et al. [22], and the PCR program described in Perrigo et al. [23]. A BLASTn search was performed using the National Center for Biotechnology Information (NCBI) GenBank database to identify the most similar SSU sequences and confirm the genus-level placement of the new species. Search results indicated that all five new species are members of *Cavenderia.* SSU sequences for all closely related species were downloaded from GenBank (Table 2) for phylogenetic analysis. Newly generated sequences were deposited in GenBank: accession numbers are available in Table 2.

**Table 2:** NCBI GenBank accession information for ribosomal SSU sequences of all isolates included in the phylogenetic analysis. New species are indicated in bold. Outgroup taxa are indicated with asterisks. All isolates noted in this table are freely and publicly available from DictyBase (Northwestern University, Evanston, IL, USA) through dictybase.org

Taxon	Isolate no.	Accession no.
*Cavenderia amphispora* ^*^	BM9A	HQ141521
*C. antarctica*	NZ43B	AM168080
***C. aureostabilis***	TH10B	MH745571
*C. aureostipes*	JKS150	AM168082
*C. aureostipes var. helvetia*	GE1	AM168095
***C. bhumiboliana***	TH11CX	HQ141523
*C. bifurcata*	UK5	AM168084
*C. delicata*	TNS226	AM168093
*C. exigua* ^*^	KP94	AM168085
*C. fasciculata*	SH3	AM168087
*C. granulophora*	CH11–4	AM168072
*C. medusoides*	OH592	AM168088
*C. mexicana*	Mex-TF4B1	AM168089
*C. myxobasis*	NT2A	HQ141522
*C. parvispora* ^*^	OS126	AM168091
***C. protodigitata***	TH18BA	MH745572
***C. pseudoaureostipes***	TH39A	HQ141518
*C. stellata*	SAB7B	AM168081
***C. subdiscoidea***	TH1A	HQ141515

SSU phylogenies were built using GenBank sequence data from the genus to determine their phylogenetic relationships with other taxa in the group. Ingroup and outgroup taxa were selected based on an earlier molecular investigation of the *aureostipes*-complex [13].

The SSU sequences of the five new taxa were aligned with sequences from 11 closely related taxa and a three further outgroup taxa from the same genus (Table 2). A multiple sequence alignment was performed with MUSCLE [24] in AliView v.1.19 [25] and then manually adjusted and trimmed. A maximum likelihood phylogeny was then inferred using RAxML HPC Blackbox and a Bayesian phylogeny was inferred using XSEDE, both with default settings and on the Cipres portal [26]. Manual inspection of the resulting phylogenies’ topologies indicated no well-supported incongruences between the results from the two methods (with a cut-off of maximum likelihood bootstrap support (BS) over 50% and Bayesian inference posterior probabilities (PP) over 0.70).

## Abbreviations

BS: Bootstrap support
L: Large
M: Median
NCBI: National Center for Biotechnology Information
PCR: Polymerase chain reaction
PG: Polar granules
PG +: With polar granules present in the spores
PP: Posterior probabilities
PV: *Polysphondylium violaceum* type of aggregation
S: Small
SSU: Ribosomal small subunit
